# Residents’ perspectives of mobile X-ray services in support of healthcare-in-place in residential aged care facilities: a qualitative study

**DOI:** 10.1186/s12877-022-03212-2

**Published:** 2022-06-25

**Authors:** Joanne Dollard, Jane Edwards, Lalit Yadav, Virginie Gaget, David Tivey, Maria Inacio, Guy Maddern, Renuka Visvanathan

**Affiliations:** 1 Adelaide Geriatrics Training and Research With Aged Care (GTRAC) Centre, Adelaide Medical School, Faculty of Health and Medical Sciences, University of Adelaide, 37a Woodville Rd, Woodville South, South Australia 5011 Australia; 2 Basil Hetzel Institute for Translational Health Research, Central Adelaide Local Health Network, 37 Woodville Rd, Woodville South, South Australia 5011 Australia; 3 Surgical Specialties, University of Adelaide, The Queen Elizabeth Hospital, Woodville, South Australia Australia; 4 Present Address: Royal Australasian College of Surgeons, Adelaide, South Australia Australia; 5 Registry of Senior Australians, South Australian Health and Medical Research Institute, Adelaide, South Australia Australia; 6 UniSA Allied Health and Human Movement, University of South Australia, Adelaide, South Australia 5001 Australia; 7 Aged and Extended Care Services, The Queen Elizabeth Hospital, Central Adelaide Local Health Network, Adelaide, Australia

**Keywords:** Nursing homes, X-ray, Hospitals, Delivery of healthcare, Healthy ageing

## Abstract

**Background:**

Mobile X-ray services (MXS) could be used to investigate clinical issues in aged care residents within familiar surroundings, reducing transfers to and from emergency departments and enabling healthcare to be delivered in residential aged care facilities. There is however little research exploring consumer perspectives about such services. The objective of this research was to explore the perspectives and preferences of residents about the provision of MXS in residential aged care facilities, including their knowledge about the service, perceived benefits, and factors that require consideration for effective implementation.

**Methods:**

A qualitative study design was used. The setting for the study included four residential aged care facilities of different sizes from different parts of a South Australian city. Purposive sampling was used to recruit participants. 16 residents participated in semi-structured interviews that were audio-recorded and transcribed verbatim. Data were inductively derived using thematic analysis.

**Results:**

Participants had a mean age of 85 years, 56% were female, 25% had dementia and 25% had had a mobile X-ray in the last 12 months. Four themes were developed. Participants preferred mobile X-rays, provided as healthcare-in-place, to improve accessibility to them and minimize physical and psychological discomfort. Participants had expectations about the processes for receiving mobile X-rays. Costs of X-rays to people, family and society were a consideration. Decision making required residents be informed about mobile X-rays.

**Conclusions:**

Residents have positive views of MXS as they can receive healthcare-in-place, with familiar people and surroundings. They emphasised that MXS delivered in residential aged care facilities need to be of equivalent quality to those found in other settings. Increased awareness of mobile X-ray services is required.

**Supplementary Information:**

The online version contains supplementary material available at 10.1186/s12877-022-03212-2.

## Background

Currently in Australia the demand for long-term residential care for people unable to live independently translates to 830 government approved residential aged care providers, delivering care to 143,117 residents [1] in residential facilities (or nursing homes [2]).

Because people are delaying entry into residential aged care facilities (RACF), given that they prefer living at home longer, and governments are also supporting ageing-in-place initiatives, people at the time of assessment for permanent entry to residential aged care facilities have increasingly complex health care needs [3]. For example, the frailty levels of older adults assessed for residential aged care more than doubled from 32% in 2003 to 75% in 2013 [4]. Furthermore, almost two-thirds of residents have dementia as a co-morbidity [5].

Evidence is emerging that more healthcare-in-place is desirable. Remaining in place is less stressful and safer for aged care residents [6], and reduces their exposure to hospital acquired complications, such as infections, falls and functional decline [7, 8]. Furthermore, the Australian Medical Association estimates that as many as 27,000 hospital admissions in the 2020–21 financial year were potentially preventable. This represents a cost of 160,000 patient bed days and AUD$312 million [9]. Transfers to emergency departments without admission added an estimated AUD$112 million [9]. Models of care that support the delivery of healthcare-in-place offer potential healthcare savings, as well as more appropriate care of a vulnerable population.

Mobile X-ray technology has long been used for diagnosing and monitoring patients in different ward settings, such as intensive care units, as well as outside of hospitals in prisons and RACFs [10]. Because there are often challenges associated with transporting frail, confused and debilitated residents to hospital, the provision of mobile X-rays in aged care facilities is appealing. Private mobile X-ray services (MXS) have been available for some time in Australia, but often generate additional charges beyond the cost of the X-ray service, because of the additional costs from transportation of equipment to and from facilities. Recognizing this as a barrier to the uptake of MXS, the Australian government (through Medicare, the Australian universal health insurance scheme) introduced a subsidy (AUD $73.65) in November 2019 to subsidise transportation costs (or call-out fee) for a mobile X-ray unit. This subsidy would be in addition to the usual rebate for the cost of the X-ray service, where for example, a chest (lung fields) X-ray attracts AUD $41.10 from Medicare and from November 2019, the radiology service provider would receive a rebate for both the X-ray and the call-out fee resulting in a total rebate of AUD $114.75 as opposed to just AUD $41.10 [11]. The call-out fee subsidy is for specific indications: X-rays of extremities, shoulder, pelvis, ribs and sternum post fall, suspected pneumonia or health failure (chest X-ray) and acute abdomen or bowl obstruction (plain abdominal X-ray) [11]. However, even when more than one resident of a facility receives mobile X-ray services in a single call-out, only one rebate is payable.

A systematic review found that using mobile X-rays in RACFs reduced the transfer of residents to emergency departments, because doctors could more confidently assess and manage patients for selected health conditions in the residential facility [12]. For example, a 2015 retrospective before-after cohort evaluation of a MXS attending RACF in one Australian state (Victoria) reported an 11.5% significant reduction in ED presentations requiring chest, hip and pelvis, spine and abdomen X-rays [13]. However, a randomised controlled trial published since then (in 2020) where Danish aged care residents received either in-house mobile or hospital X-ray found no difference in subsequent hospitalisation rates (11.8% vs 12.1% respectively). The findings from this study however should not be relied upon, as the authors acknowledge that the study was underpowered and flawed, with some frail residents who were randomized to hospital X-ray withdrawn by their GP and the most frail residents treated in the nursing home without an X-ray [14]. Therefore, these data suggest that good quality evidence is needed to test whether MXS is effective in increasing hospital avoidance. Moreover, evidence is also needed from diverse settings, such as the Australian context, given the variation in local health service financing and provision, to allow definitive conclusions about the role of MXS in increasing hospital avoidance. Regardless, it is generally accepted that avoidable hospital presentation is preferred and that MXS are one within a suite of hospital avoidance programs [15, 16]. In an effort to improve hospital avoidance and aid timely diagnosis and treatment, the Australian government have introduced the subsidy to improve uptake of MXS. Such evidence should include the perspectives of residents because residents have the right to be consulted, as enshrined in the Charter of Aged Care Rights in Australia [17]. The Charter encourages service providers to incorporate resident preferences in service delivery. In addition, services are more likely to be effective if they are designed to meet consumer need, preferences, and expectations. However, no qualitative study has explored the consumer perspective of health-in-place care. One Danish study conducted an observation study with residents living with dementia receiving a mobile X-ray. They concluded that residents remaining in their own environment whilst receiving an X-ray benefited, as evidenced by their calm behaviour [6]. No studies have reported on residents’ perspectives. This study explored the perspectives and preferences of residents about the use of MXSs in RACFs in terms of residents’ knowledge about the service, perceived benefits, and factors that need to be considered if such services are to be used by residents more widely.

## Methods

### Ethics and consent

This study received ethical approval from the Human Research Ethics Committee of the University of Adelaide (Ref: H2020-197).

### Setting

Six aged care organisations from Adelaide were approached to participate in this study. Two declined and the remaining four organisations each nominated a residential aged care facility (RACF). These four RACF were geographically disparate areas across Adelaide. RACFs had a range of bed numbers (1 RACF of 50 to 100 beds; 2 RACFs 101 to 150 beds; 1 RACF 151 to 200 beds). The residents of one RACF were significantly more culturally and linguistically diverse than participants from the three other RACFs.

### Participants and recruitment

Purposeful sampling was used to recruit participants from the RACF. Staff from the residential aged care facilities recruited study participants. Inclusion criteria included residents of the selected aged care facilities who could give informed consent (as judged by RACF staff who knew residents) and could verbally communicate to engage in a face-to-face, telephone or online interview. Residents who had experienced an X-ray in the aged care facility or in an emergency department were preferred, but this was not a requirement. There were no exclusion criteria.

The residential aged care facilities were each offered a $500 honorarium for their assistance in recruiting the participants, setting up interviews and collecting participant data from resident records. The goal was to recruit five residents per participating facility (*n* = 20) or until data saturation.

Staff from the RACF provided verbal and written information about the study to residents. If residents expressed a willingness to participate, they were informed that their participation was voluntary, and that they could withdraw their consent anytime without specifying any reason. Residents gave written informed consent before being interviewed and verbally reconfirmed consent prior to the interview.

### Data collection

A review of the literature and the experience of the research team guided the development of the interview schedule (Additional File 1). The following areas were explored with residents: a) what was important to their lives; b) their knowledge of mobile X-rays; c) the pros and cons of mobile X-rays; d) the factors that should be considered; and e) their willingness to pay the call-out fee for mobile X-rays. The interview schedule was piloted on the first interview and was considered appropriate.

One researcher (JD; an experienced qualitative researcher with previous experience of working clinically and research wise with people with dementia) conducted semi-structured interviews, at a time that suited the resident (November 2020 to February 2021). RACF staff reminded residents of the interview time, assisted with setting up the telephone or device and ensured a quiet background such as turning off the television. Mindful that participants may have cognitive and or sensory impairment, interviews were conducted by speaking clearly, giving participants time to respond, being sensitive to verbal and non-verbal cues, and adapting within the interview to accommodate participant needs [18].

At the end of each interview, the interviewer summarised their understanding of what participants had said, and encouraged participants to add or correct information. Field notes were written immediately after the interview, reflecting on the interview and key points and observations. Interviews were audio-recorded and transcribed verbatim. Interviews were conducted until data saturation.

After the interviews, assisting staff from the residential aged care facilities recorded participants’ age, gender, years lived in the facility and dementia diagnosis from medical records. Staff also recorded reason and location (RACF, emergency department or community setting) of participant X-rays in the last 12 months (if the resident had been living in the RACF for less 12 months, then this period only was included).

### Analysis

Data were thematically analysed (guided by a six-stage process) of thematic analysis, looking for repeated patterns of meaning (Braun & Clarke, 2006). Transcripts were read and reread, along with field notes, and initial codes developed [19]. Themes were developed, reviewed and defined. An inductive approach was used to generate themes derived from the interviews as well as being sensitive to themes generated from the literature. Three experienced qualitative researchers (JD, JE, LY) independently coded three transcripts, to review, discuss and refine coding and interpretation. The research team met frequently to deliberate the results of the analysis. NVivo 12 was used to assist with data management and analysis. Quotations are provided as evidence to support themes. Themes and further quotes are provided in Additional File 2. Data relating to resident characteristics were entered into SPSS 28 and descriptively analysed.

## Results

Altogether, 27 residents were approached. Seven residents declined to participate, with reasons cited including: not feeling well, wanted daughter present, had recently participated in other research or not interested. The family of one resident declined to consent, but provided no reason. Aged care staff withdrew one participant whilst family withdrew another. One participant withdrew because they were unwell.

Sixteen residents were interviewed. Residents’ mean age was 85 years (range 73–95 years), and nine residents (56%) were female. One quarter of the participants (*n* = 4) had a diagnosis of dementia. Nine (56%) residents had lived in their RACF for less than 12 months.

In total, six participants had an X-ray in the last 12 months, and two participants had received two X-rays (in total, 10 X-rays). Four participants had an elective (i.e. non urgent) X-ray in the RACF, for investigation into pain unrelated to a fall (knee, low back, hip/leg) and to check cancer progression (spine). Two participants had received an X-ray in an emergency department and four had received an X-ray at a community radiology centre. One participant received an X-ray in emergency department and community radiology; one received an X-ray in RACF and community radiology. Eight participants had not received an X-ray in the last 12 months.

Interviews averaged 39 min (range 21 to 57 min). Interviews were conducted either by Zoom (*n* = 3), telephone (*n* = 4) or face-to-face (*n* = 9). Four themes were developed through data analysis. The first theme, preferring healthcare-in-place included three sub-themes: improving accessibility to X-rays, minimising physical and psychological discomfort and remaining in their comfort zone. The second theme, expectations regarding processes for mobile X-ray included three sub-themes: impact of RACF staffing, radiographer skills and image quality, and timely investigation and GP follow up. The third theme, economic, personal and society cost of mobile X-ray included two sub-themes: cost to family and society on accessing fixed X-ray and cost to resident of mobile X-ray call-out fee and equity issues. The fourth theme, awareness of mobile X-rays included two sub-themes: level of awareness and wanting increased awareness.

### Preferring healthcare-in-place

Many participants expressed an enthusiastic preference for healthcare, such as mobile X-rays, to be delivered in their place of residence, for themselves and other residents. Participants noted that there were times when the severity or urgency of their illness might indicate that investigation or treatment in hospital was necessary. In these circumstances, their preference would depend on the situation.

*Well, to go in hospital is good if you have a very, very bad condition, but if you avoid going in hospital, is much better [RACF A; ID01]*

Participants viewed MXSs as improving residents’ accessibility to X-ray, especially when residents had mobility impairments and pre-existing health conditions which impacted their wellbeing and ability to easily access health services. Mobile X-rays minimised the disruption and physical and psychological discomfort entailed in leaving the RACF.

*Some people can’t get out and about so they need something that can be brought to them or they can get to it somehow [RACF C; ID25]*

*It saves me going in an ambulance or Access Cab. […]. I'd have to get a carer to go with me. Maybe they have to get me up on a table. So they have to do that. They've got to get a sling, which I've got a sling I take with me and things like that. Lift me onto the table, roll me around all over the place and in general, it could be quite - well for me, it's quite exhausting actually. […] The reason is it's just my personal comfort. […] Got to have personal comfort.* That's about *all I've got going for me now [RACF B; ID10]*

*I reckon they should come. Elderly people should not go to the hospital to get one [an X-ray]; they should come to the elderly people, I reckon. That's better for them because elderly people, they suffer a lot [RACF B; ID08]*

*Oh that was just plain luxury […] I’m not very mobile and to get in a taxi and go off to another hospital and have X-rays - with the walking around it would have been very difficult. So it saved me all that [RACF C; ID26]*

Having to go to hospital or community radiology for an X-ray can increase resident physical and psychological discomfort. Participants talked of being traumatised by their experience, including having to be transported, waiting, and feeling out of place, isolated, overlooked and uncomfortable.

*Because it saves you a trip to hospital and sitting around for hours on end [RACF A; ID03]*

*Not comfortable, hm, and quite often if you're in emergency […], they plonk you in a place and people walk past you and stare at you and oh dear [RACF B; ID06]*

*One time I went […] to the […] hospital for an X-ray and I waited five hours for an X-ray. I was very very tired and sore. I reckon especially for people with bad backs, it doesn't matter, elderly people, it's not too good, waiting all that time [RACF B; ID08]*

*Oh, I would love it. I really would, not having* to go out and go through that trauma, because if you go to any clinic […]. You can sit there for a couple of hours before it's your turn, but it's such a waste of *time, and tedious [RACF B; ID07]*

Participants also valued the opportunity to have a mobile X-ray in their own familiar room with minimal disruption to their routines, activities, comforts and additionally feel in more control of how they use their time waiting during the process.

*It gets you right down to the heart of the problem I gather. That fact that you can do it without moving out of your room is something which is supremely important [RACF C; ID26]*

*Because I can look after myself in my own room. I can be content in my own room. […] I can make—keep myself busy [RACF B; ID06]*

*Because if it's here, then I can wait for the results in familiar surroundings and not have the trouble of other people around me I don't know, strange surroundings, that sort of thing, just to have - to be in my own room in familiar surroundings, with people I know and care about. So like I said, I'm all for it [RACF B; ID07]*

*I can still watch television [RACF B; ID10]*

While avoiding the discomfort of transfer for an X-ray, one participant who received a mobile X-ray reported the examination was briefly painful, due to their impaired mobility.

*The only painful bit about it was that I had to lie on the board at one stage as you would realise. When you’re lying on your side on a board with a rather stiff, elderly body, it gets a little painful. But it didn’t take very long [RACF C; ID26]*

### Expectations regarding processes for mobile X-ray

Participants had expectations regarding the processes for having a mobile X-ray and considered potential issues that might impact on its implementation. Some participants emphasised that staffing constraints in the RACF could be a limitation to effective delivery of healthcare-in-place, such as mobile X-ray.

*I think to a certain degree you've got to rest on the staffing […] remembering, of course, that the staffing in any nursing home, it's changing every day. There's no consistency. There's no carryover. That's where nursing homes fall down. There's a lack of consistency in the management [RACF C; ID30]*

Participants expected that staff conducting the mobile X-ray would need technical skills in radiography and also required skills such as empathy, communication (particularly for people with dementia), clear English and good manual handling to position residents who might be difficult to position.

I think the people you send along with the machine. Because a little bit of empathy goes a long way. You are not dealing with normal people. Some might have dementia. Some might have some *other things. You've got to be pretty careful how you handle them. Because if you don't handle them right, you have them screaming and carrying on […] Just get the right people for the right job [RACF B; ID10]*

Participants expected X-rays to have good quality images and to be undertaken and reported in a timely manner, regardless of location.

*So, I take it [assume], it took as clear a picture as an ordinary X-ray […] My first response was, well I hope it works [RACF C; ID26]*

*If there's a mobile man available and he's coming within a reasonable amount of time, I'd say go for it. If he can't come or they can't get anybody or they've rung the mobile man and he's not available, […] well, then you go to the hospital and the X-ray department there or whatever [RACF C; ID30]*

Another expectation was the follow up from the GP following the X-ray.

*As I say, the doctor who apparently arranged the appointment fell ill for some reason. […] I have really not much idea of […] what the X-ray uncovered [RACF C; ID26]*

### Economic, personal and societal cost of mobile X-ray

Participants were concerned about the costs to their families when they needed to be transported out of the facility for X-rays. They described these costs in terms of loss of income, loss of time and inconvenience. It was also acknowledged there was a cost to the RACF if a carer had to be provided.

*If I had to go for an X-ray, […] my son would insist to take me wherever I had to go, […] which would mean he would have to take a day off and lose his income for that day. No matter what I said, he would insist [RACF B; ID07]*

*No. I haven’t got any family in the state. So that’s a bit difficult. I would have had a carer from the home would have gone with me as an escort [RACF C; ID26]*

Some participants did not want to pay for a call-out fee. Other participants did not personally view a call-out fee for MXSs as an issue for them, but expressed concerns for others, such as those on an aged pension or from lower socio-economic backgrounds.

*Well, most of the people in the place here would be the same. They'd be pensioners who go out and get things free anyway. They would turn around and think of the cost I think, because if you're on a pension you're watching your pennies [RACF B; ID06]*

### Awareness of mobile X-ray

Many participants were unaware that MXSs existed, much less that they were available at their RACF. If they were aware of mobile X-ray technology, it was usually because of their experiences in hospital, or they had heard about it from someone else who had read advertising material or information on the RACF notice board.

*Actually, […] I knew about mobile X-rays because they use them in the hospitals when they have big operations - you know, your hip and knee operations - after that they always bring a mobile X-ray in and X-ray you. [...] Instead of moving the patient, they bring the machine…but I didn’t know about going out into the - into anywhere actually [RACF B; ID06]*

*Virtually nothing. That they are mobile [RACF A; ID03]*

Participants suggested solutions, such as making information about access to mobile X-ray services, along with its benefits, available in RACF via staff or written information. Some of the participants who had experienced a mobile X-ray promoted them to other residents.

I like to know and forearmed is forewarned and all that sort of stuff. *I like to know, […] and I did ask questions about it at the time when I saw it [brochure],* ‘Is this available?’ It [brochure] wasn’t *there for very long. I had a couple of people come to me after that, not because they’d seen the brochure but because they needed to have X-rays. I said ‘Are you aware you don’t have to go out to have* it? You can have it come to you.’ ‘Oh really’, they said. […] ‘Yes, ask. It is available.’ *[RACF C; ID29]*

*Information sheets. I think every nursing home should have a booklet setting out all the facilities that are available in that particular nursing home, so that when people come in for the first time as new people moving in, they are given the information with all the information that they may need […] or that they could use in an emergency. Even though they're not using it at the time and may never use it, they need to know that it is available [RACF C; ID30]*

## Discussion/conclusion

The feelings of comfort and safety from remaining in a familiar environment noted in this exploratory research were similar to those observed in a study by Jensen [6]. Residents supported the use of MXSs and preferred to avoid hospitals [6]. However, they recognized that sometimes transfer to hospital was necessary, either because of clinical need or staffing issues within facilities. A recent qualitative study from Switzerland explored the perspectives of residents and carers in relation to changes in resident health while in a residential aged care facility [20]. Like our research, residents in the Swiss study expected that personal and relationship needs would be met as part of acute care management [20]. The Swiss researchers described resident and family perceptions of the limitations of staff skills and availability, referring to ‘an orchestra playing its standards’ [20]. Participants in that study also noted that skilled staff and access to general practitioners were not guaranteed in residential aged care facilities, which reduced the likelihood of an acute episode of ill health being adequately managed within the facility [20].

As with the Swiss study, participants in our study understood that in some acute situations the RACF had reached its limits [20] and a transfer to hospital was necessary. Although they preferred to remain in their RACF, participants did not want their health treatment compromised. This applied not only to the timeliness and quality of X-ray services but also the timeliness and quality of subsequent treatment. Reassuringly, a recent scoping review suggests that the image quality mobile X-ray technology is good [10]. However, services to support healthcare-in-place, in addition to those routinely provided in facilities, are required to ensure timely, safe and quality acute care, in line with resident expectations. MXS, along with other hospital avoidance strategies, are ways of supporting facilities so that healthcare in place is achievable.

In the Swiss study, it was mentioned that when residents were acutely ill, ‘the audience compensates for orchestra limitations’ [20]. Our study also revealed a high level of support for one another among the residents and staff, and highlighted the increased burden on informal carers that hospital transfers or moves to radiology centres engendered. When away from familiar residents and staff, ill or injured residents had to rely on family or friends for support. If clinical services, such as the taking of X-rays, were more available in RACFs, this burden on others would be lessened.

Perceptions of being a burden can adversely affect resident wellbeing, increasing feelings of helplessness [21], while family and friends experience a sense of increased responsibility for the resident. Health services often expect informal carers to provide direct care while navigating complex health systems on behalf of the resident [21]. This imposes a heavy, sometimes unsustainable, impost on carers.

Participants in the current study revealed that both physical and cognitive vulnerabilities influenced their wellbeing. This accords with findings by Jensen and colleagues who found that interactions around X-ray procedures in RACF differed with the resident’s level of dementia, and both verbal and non-verbal communication between residents and radiographers could be difficult [6]. Given that residents in this study acknowledged the importance of cognitive vulnerability in negotiating various dimensions of healthcare, our study supports the findings of Jensen et al. that radiographers needed more than technological competence; they needed excellent communication skills for dealing with cognitively vulnerable residents [6]. Training that provides gerontology skills and competence in managing older people with frailty and dementia is likely to augment the perceived efficacy of MXS in a residential aged care setting and increase the desirability of the service.

The call-out fee subsidy was developed to encourage the use of MXSs [11]. However, the 25% of our sample who received a mobile X-ray in the last 12 months were ordered an X-ray for reasons which were in-eligible for the call-out fee subsidy. This suggests that MXSs could have a role in the care of residents, extended beyond hospital avoidance and beyond the limited X-ray services that is covered by the subsidy for the call-out fee by the Australian government. Further, it is evident greater awareness of the service is also required, according to the study data, to potentially increase use of mobile X-rays. Knowledge of what can be provided and its benefits needs to be widely disseminated to encourage uptake of mobile X-ray technology. In a recently published qualitative study from Australia exploring the consumer experience in relation to a community-based hospital avoidance program, the authors noted that at the commencement of the service, consumers had limited knowledge of the program and recommended the provision of more information as an area of service improvement [22]. Being aware that the service exists and being aware of how it can be used empowers residents and better ensures their active engagement in the decision-making process relating to their healthcare within and external to the RACF.

A major strength of this study was the involvement of residents (including some with dementia) from multiple facilities and varied aged care organisations in terms of size, location and cultural backgrounds. The study was conducted in one state in Australia and further research in other health jurisdictions is likely to produce other relevant perspectives. As 56% of participants had lived in their RACF for less than 12 months, X-ray experience was not collected about residents experience with X-rays beyond them living in their RACF. In addition, only a small number of participants in this study had personally experienced a mobile X-ray, with none of these for emergency circumstances and so further exploration with a larger sample of residents having experienced a MXS in RACF is necessary, which is underway.

It is now well established, that patient-centred interventions, can only be developed by incorporating residents’ first-hand insights into their experience of the care they receive and the circumstances and environments in which they receive it [23]. This research therefore adds to our understanding of the perspectives and expectations of residents in relation to the delivery of mobile X-ray services in residential aged care facilities. This could help pave the way for better delivery of this technology and thus improve healthcare for a vulnerable population. It will also be important to include the views of other stakeholders, and we have explored the perspectives of informal caregivers (manuscript in preparation) and stakeholders in health care and aged care (manuscript in review, BMC Geriatrics 2022).

There is support from residents for the use of MXS in RACF because they can receive healthcare in a place where they feel secure and are with familiar people, reducing the burden on informal carers. What residents want from MXS can be drawn on to design MXS services that are acceptable to residents and may lead to increased hospital avoidance, where appropriate, which residents value. Finally, MXS services can benefit the entire health care system by alleviating pressure on the acute care system. Other in-reach services used to increase hospital avoidance, such as acute geriatric services should be designed and evaluated with input from the views of residents, so that research has broader applicability [24].

## Supplementary Information

**Additional file 1.**

**Additional file 2.**

## Data Availability

Requests for data should be directed to the corresponding author (joanne.dollard@adelaide.edu.au) and ensuing research will require collaboration with the chief investigators (RV and JD). Any requests will be assessed for scientific rigor by this investigator team. A request for ethics approval and/or amendment must be prepared by the requestor in line with the requirements of Human Research Ethics Committee of the University of Adelaide. This ethics approval/amendment must be approved by the Human Research Ethics Committee of the University of Adelaide. A data sharing agreement will need to be put in place. The requestor will be responsible for providing the necessary funding required for this process, including for the provision of data. Given that the grant funding for the project is in place to the 31st of December 2022 and there may be analyses continuing, then data sharing is embargoed till the 30th of March 2023.

## Abbreviations

GP: General practitioner
MXS: Mobile X-ray service
RACF: Residential aged care facility

## References

[1] Department of Health (2021). 2020–21 Report on the Operation of the Aged Care Act 1997.

[2] Sanford AM, Orrell M, Tolson D, Abbatecola AM, Arai H, Bauer JM, et al. An international definition for “nursing home.” J Am Med Dir Assoc. 2015;16(3):181–4. 10.1016/j.jamda.2014.12.013 25704126

[3] Australian Institute of Health and Welfare. Australia’s welfare 2015 Canberra: AIHW. https://www.aihw.gov.au/getmedia/692fd1d4-0e81-41da-82af-be623a4e00ae/18960-aw15.pdf.aspx?inline=true. Accessed 7 Mar 2022.

[4] Khadka J, Visvanathan R, Theou O, Moldovan M, Amare AT, Lang C (2020). Development and validation of a frailty index based on Australian aged care assessment program data. Med J Aust.

[5] Jadczak AD, Robson L, Cooper T, Bell JS, Visvanathan R, Karnon J (2021). The Frailty In Residential Sector over Time (FIRST) study: methods and baseline cohort description. BMC Geriatr.

[6] Jensen JM, Andersen PAB, Kirkegaard L, Larsen N, Most W, Nielsen D, et al. Exploring the patient perspectives of mobile X-ray in nursing homes - A qualitative explorative pilot study. Radiography (London, England : 1995). 2021;27(2):279–83. 10.1016/j.radi.2020.08.009 32919898

[7] Walsh EG, Wiener JM, Haber S, Bragg A, Freiman M, Ouslander JG (2012). Potentially avoidable hospitalizations of dually eligible Medicare and Medicaid beneficiaries from nursing facility and Home- and Community-Based Services waiver programs. J Am Geriatr Soc.

[8] Guilcher SJT, Everall AC, Cadel L, Li J, Kuluski K (2021). A qualitative study exploring the lived experiences of deconditioning in hospital in Ontario, Canada. BMC Geriatr.

[9] Australian Medical Association. AMA identifies savings of $21.2 billion in aged care hospital admissions [press release]. 2021. https://www.ama.com.au/media/ama-identifies-savings-212-billion-aged-care-hospital-admissions. Accessed 7 Mar 2022.

[10] Toppenberg MD, Christiansen TEM, Rasmussen F, Nielsen CP, Damsgaard EM (2020). Mobile X-ray outside the hospital: a scoping review. BMC Health Serv Res.

[11] Department of Health. New MBS Item for mobile provision of skeletal X-ray to patients within residential aged care facilities factsheet. 2019. http://www.mbsonline.gov.au/internet/mbsonline/publishing.nsf/Content/Factsheet-MobileXray. Accessed 11 May 2022.

[12] Kjelle E, Lysdahl KB (2017). Mobile radiography services in nursing homes: a systematic review of residents’ and societal outcomes. BMC Health Serv Res.

[13] Montalto M, Shay S, Le A (2015). Evaluation of a mobile X-ray service for elderly residents of residential aged care facilities. Aust Health Rev.

[14] Toppenberg M, Christiansen T, Rasmussen F, Nielsen C, Damsgaard EM (2020). Mobile X-ray outside the hospital vs. X-ray at the hospital Challenges exposed in an explorative RCT study. Healthcare.

[15] Testa L, Ryder T, Braithwaite J, Mitchell RJ (2021). Factors impacting hospital avoidance program utilisation in the care of acutely unwell residential aged care facility residents. BMC Health Serv Res.

[16] Loeb M, Carusone SC, Goeree R, Walter SD, Brazil K, Krueger P (2006). Effect of a clinical pathway to reduce hospitalizations in nursing home residents with pneumonia: a randomized controlled trial. JAMA.

[17] Australian Government Aged Care Quality and Safety Commission. Charter of Aged Care Rights. https://www.agedcarequality.gov.au/consumers/consumer-rights#charter-of-aged-care-rights. Accessed 9 May 2022.

[18] Webb J, Williams V, Gall M, Dowling S. Misfitting the research process: shaping qualitative research “in the field” to fit people living with dementia. Int J Qual Methods. 2020;19:1–11.

[19] Braun V, Clarke V (2006). Using thematic analysis in psychology. Qual Res Psychol.

[20] Basinska K, Künzler-Heule P, Guerbaai RA, Zúñiga F, Simon M, Wellens NIH, et al. Residents’ and relatives’ experiences of acute situations: a qualitative study to inform a care model. Gerontologist. 2021;61(7):1041–52. 10.1093/geront/gnab027 PMC8437505 33624766

[21] Lilleheie I, Debesay J, Bye A, Bergland A. The tension between carrying a burden and feeling like a burden: a qualitative study of informal caregivers’ and care recipients’ experiences after patient discharge from hospital. Int J Qual Stud Health Well-being. 2021;16(1):1855751. 10.1080/17482631.2020.1855751 PMC7758041 33345749

[22] Pereira RB, Brown TL, Guida A, Hyett N, Nolan M, Oppedisano L (2021). Consumer experiences of care coordination for people living with chronic conditions and other complex needs: an inclusive and co-produced research study. Aust Health Rev.

[23] O’Cathain A, Croot L, Duncan E, Rousseau N, Sworn K, Turner KM, et al. Guidance on how to develop complex interventions to improve health and healthcare. BMJ Open. 2019;9(8):e029954. 10.1136/bmjopen-2019-029954 PMC6701588 31420394

[24] Dai J, Liu F, Irwanto D, Kumar M, Tiwari N, Chen J (2021). Impact of an acute geriatric outreach service to residential aged care facilities on hospital admissions. Aging Med (Milton).

