# Supplementary material for: Residents’ perspectives of mobile X-ray services in support of healthcare-in-place in residential aged care facilities: a qualitative study
Source: BMC Geriatr. 2022 Jun 25;22:525. doi: 10.1186/s12877-022-03212-2 (PMC9233760; doi:10.1186/s12877-022-03212-2)
Supplement: Supplementary file 2 — Additional file 2. [file 12877_2022_3212_MOESM2_ESM.docx]

**Additional File 2:** Themes and supporting quotes

| Themes | Sub-themes | Example quotes |
| --- | --- | --- |
| Preferring healthcare-in-place | Improving accessibility to X-rays | I think it's an extremely remarkably good offer. […] It is because there are a lot of people involved in getting me into a chair, getting me pushed and so on and so forth. Whereas, if you came to give me one [X-ray], I assume the most I would do would be shift from my chair onto whatever you required. [Otherwise] after I'd been dealt with, which is usually after a long delay, I would be pushed back to the car, and by this time I'd be gasping like anything.[…] COPD; I can't breathe [RACF D; ID 17]. |
|  | Minimising physical and psychological discomfort | For everything, save a lot of time, a lot of time and for the people is much better because when you go in hospital, I know when many time[s] myself and it’s very hard. It’s very hard. You go there, they put you there, then okay the X-ray, they you do give straightaway and then you wait. When you finish everything they got to call an ambulance after the doctor said you can go home. Sometime[s] you can wait four hour[s], sometimes you got to stay all night because there’s no ambulance in the night. […] It is way too hard to stay there because you’re not in a comfortable bed. [RACF A; ID02] |
|  | Remaining in their comfort zone | You think of what that's going to do to him to be dragged off to [hospital] or wherever he has to go. Wouldn't it be much better for him to be lying here on his own bed? [RACF C; ID30]. |
| Expectations regarding processes for health investigation | Impact of RACF staffing | You've got to remember, they've got work to do as well. […] It depends when it's done. But you wouldn't want to come in the morning. Well, at least until after 11 o'clock. […] Maybe 10:00 am. [RACF B; ID10]. |
|  | Radiographer skills and quality imaging | I imagine these people would all be trained experts [RACF C; ID29].  I would trust you that it would be as good as in a hospital. [RACF D; ID17]. |
|  | Timely investigation and GP follow up | Well, the only thing or condition I could think of would be if I fell off my chair and broke my leg, for example. Well, if it were a week day and you were able to come right away, that would be preferable to ringing the ambulance to take me to hospital. But I don't know how long you react [RACF D; ID17]. |
| Economic, personal, and societal cost of mobile X-ray | Cost to family and society on location based X-ray | When you've got relatives who are in very, very important jobs, professional people, who have to leave their job to come and help you do something, you're very glad to let them off the hook and make sure that they don't have to come [RACF C; ID30]. |
|  | Cost to resident of mobile X-ray call out fee and equity | Well, it would be, and I'd be willing to pay for it, because I've got the finances there. But for other people that don't have that secured thing, then it might be a bit of a problem for them [RACF B; ID07]. |
| Awareness of mobile X-ray | Level of awareness | No, I don’t know much about mobile X-ray. […] No, no, I never heard that. I know the X-ray because I went many time [RACF A; ID02]. |
|  | Wanting increased awareness | I made sure that everybody around me knew about it. I said if you ever had to have an X-ray, this is what I would recommend that you do [RACF C; ID30]. |
